# Epstein-Barr virus infection shapes the genetic, transcriptomic, and immune microenvironment landscape of Burkitt lymphoma

**DOI:** 10.1186/s13027-025-00720-9

**Published:** 2025-12-10

**Authors:** Qing Xiao, Yilei Zhang, Zhongyi Chen, Xinrong Liu, Wentao Yu, Tiansheng Wang, Ling Chu

**Affiliations:** 1 Department of Pathology, The Third Xiangya Hospital, Central South University, Changsha, Hunan P.R. China; 2 Department of Otolaryngology, Head & Neck Surgery, The Third Xiangya Hospital, Central South University, Changsha, Hunan P.R. China

**Keywords:** Burkitt lymphoma, EBV, Gene alteration, Macrophage

## Abstract

Burkitt lymphoma (BL) is a highly aggressive B-cell lymphoma with its occurrence and progression closely associated with Epstein-Barr virus (EBV) status. However, the molecular differences between EBV-positive and EBV-negative BL have not been comprehensively evaluated. Through targeted sequencing of a 475-gene panel in 27 BL cases, and RNA-seq and LncRNA-seq analyses of 25 cases, we found that EBV-negative tumors displayed a higher frequency of chromosomal amplifications, particularly at 7p12.2, 6q25.3 and 7q22.1. Two novel variants of *KMT2D* were identified in adult patients in the EBV-negative group. Furthermore, EBV-positive BL exhibited enriched *FOXO1* mutations, in contrast to *CCND3* hotspot variants in EBV-negative cases. Transcriptome profiling revealed 1,612 differentially expressed genes (DEGs), predominantly involved in the PI3K-AKT, Hippo, WNT, and mTOR pathways. LncRNA profiling identified three EBV-associated lncRNAs ((MSTRG.103766.1, MSTRG.33752.11, ENSTO0000400385.2) with potential prognostic relevance in BL. Immune deconvolution (CIBERSORT/xCELL) demonstrated elevated M1 macrophages infiltration in EBV-positive BL, which correlated with improved 24-month overall survival (68% vs. 41%, *p* = 0.02). SULT1C2P1 and KCNK5 emerged as M1-associated prognostic biomarkers. Our findings establish EBV as a key modulator of BL genomic instability and immune remodeling, leading us to hypothesize that EBV status defines distinct BL subtypes with unique therapeutic vulnerabilities, thereby enabling the future development of EBV-stratified precision therapies.

**Supplementary Information:**

The online version contains supplementary material available at 10.1186/s13027-025-00720-9.

## Introduction

Burkitt lymphoma (BL) is a highly aggressive form of non-Hodgkin lymphoma (NHL), accounting for 3%-5% of all NHL cases. It is most common in children and young adults in China and frequently affects the jawbone, craniofacial bones, abdominal organs, and central nervous system [1]. A hallmark of BL is the extremely high proliferation rate of tumor cells, which has long been a research focus and a significant challenge in understanding its pathogenesis.

Genetic alterations are fundamental to the development and specific biological functions of BL. BL was the first lymphoma described to harbor a recurrent chromosomal abnormality: a balanced translocation between 8q and 14q [2]. Subsequently, rearrangement and translocation of the *MYC* gene have been confirmed to play a significant role in BL proliferation [2–4]. Although over 90% of cases involve *MYC* gene rearrangements, *MYC* dysregulation alone is usually insufficient to drive lymphomagenesis. It is often accompanied by other genetic alterations that collectively contribute to BL pathogenesis [5].

Emerging evidence suggests that gene alterations induced by Epstein–Barr virus (EBV) infection may be a critical factor in BL development and progression. EBV was discovered in Burkitt lymphoma cells in 1964 and plays a crucial role in B cell immortalization [6]. BL has three recognized subtypes: sporadic, endemic, and immunodeficiency-associated, with the occurrence of the endemic subtype being closely related to EBV infection [7]. The proportion of EBV detected in sporadic BL tumors is approximately 20–30%, as reported in studies from China and other sporadic regions [8, 9], while it is markedly higher in endemic (>95%) and immunodeficiency-associated (25–45%) BL [5]. Researches indicate that EBV status is one of the main determinants of the genetic subtypes and incidence of BL [1, 10]. In EBV-negative cases, *SOX11* expression is more likely to be associated with mutations in *SMACAR4* and *ID3* [11]. Compared to EBV-negative BL, EBV-positive tumors exhibit a distinct genetic landscape characterized by a paradoxical combination of a higher overall genomic mutation burden and fewer oncogenic driver gene mutations, highlighting the significant role of EBV in modulating the molecular genetic landscape of BL. The elevated mutation load is primarily driven by upregulation of AICDA (Activation-Induced Cytidine Deaminase), which mediates aberrant somatic hypermutation, leading to an accumulation of mutations in the non-coding regulatory regions of genes such as the *PVT1* promoter, the *PAX5* enhancer, as well as *BCL6*, *MYC*, and *ID3*. However, at the driver gene level, EBV-positive tumors show significant selectivity: they are enriched for mutations in *IGLL5* and *BACH2* but have a significantly lower mutation frequency in key apoptosis pathway genes like *TP53* and *USP7*. Furthermore, mutations in genes such as *SMARCA4* and *CCND3* are also less frequent in EBV-positive compared to negative tumors [4, 12]. These findings underscore the profound impact of EBV infection on the BL genome, which may also influence treatment responses [13]. Distinguishing between EBV-positive and negative BL is therefore essential for understanding the disease’s variability and biological heterogeneity.

However, existing studies have yet to fully elucidate how EBV infection drives BL progression through specific genetic mutations, particularly in direct comparisons between EBV-positive and EBV-negative BL. EBV is highly immunogenic, and the tumor immune microenvironment (TIME) plays a critical role in the progression of both solid tumors and hematopoietic malignancies. Numerous studies have demonstrated that EBV infection significantly modulates the TIME. In intrahepatic cholangiocarcinoma and gastric cancer, EBV-associated tumors exhibit substantial alterations in their immune composition and a significantly increased density and proportion of CD8 + T cells [14, 15]. In classical Hodgkin lymphoma, EBV-positive tumors are associated with a high density of PD-L1–positive macrophages and display a characteristic T-helper 1 (Th1) anti-tumor immune signature, marked by increased CD8 + T-cell infiltration and coordinated expression of the canonical Th1 transcription factor T-bet (TBX21), interferon-gamma (IFNG), and the IFN-γ-induced immunosuppressive enzyme indoleamine 2,3-dioxygenase [16, 17]. The TIME is a critical player in lymphoma pathogenesis, yet how EBV infection sculpts the TIME in BL remains poorly characterized, particularly in direct comparisons between EBV-positive and negative cases.

To comprehensively delineate how EBV infection drives BL progression through specific genetic mutations and immune microenvironment remodeling, we conducted integrated DNA and RNA sequencing analyses on a cohort of BL patients. This study aims to compare the genomic, transcriptomic, and immune landscapes of EBV-positive and negative BL to identify key molecular drivers and potential therapeutic vulnerabilities. Our study identified several key genes implicated in BL tumorigenesis, including *MYC*, *DDX3X*, *ID3*, and *TP53*. EBV-negative tumors displayed a higher frequency of chromosomal amplifications, particularly at 7p12.2, 6q25.3 and 7q22.1 and two novel variants of *KMT2D* were identified in adult patients of the EBV-negative group. Notably, significant differences were observed in the mutation frequencies of *FOXO1* and *CCND3* between EBV-positive and EBV-negative BL patients. Transcriptomic analysis revealed DEGs enriched in critical signaling pathways such as PI3K-AKT, Hippo, WNT, and mTOR. Importantly, DEGs between EBV-positive and negative BL overlap with survival prognostic genes. Additionally, immune infiltration analysis identified nine genes whose increased expression correlated with higher levels of M1 macrophages and NK cells, which are associated with better prognosis.

## Materials and methods

### Sample collection and selection


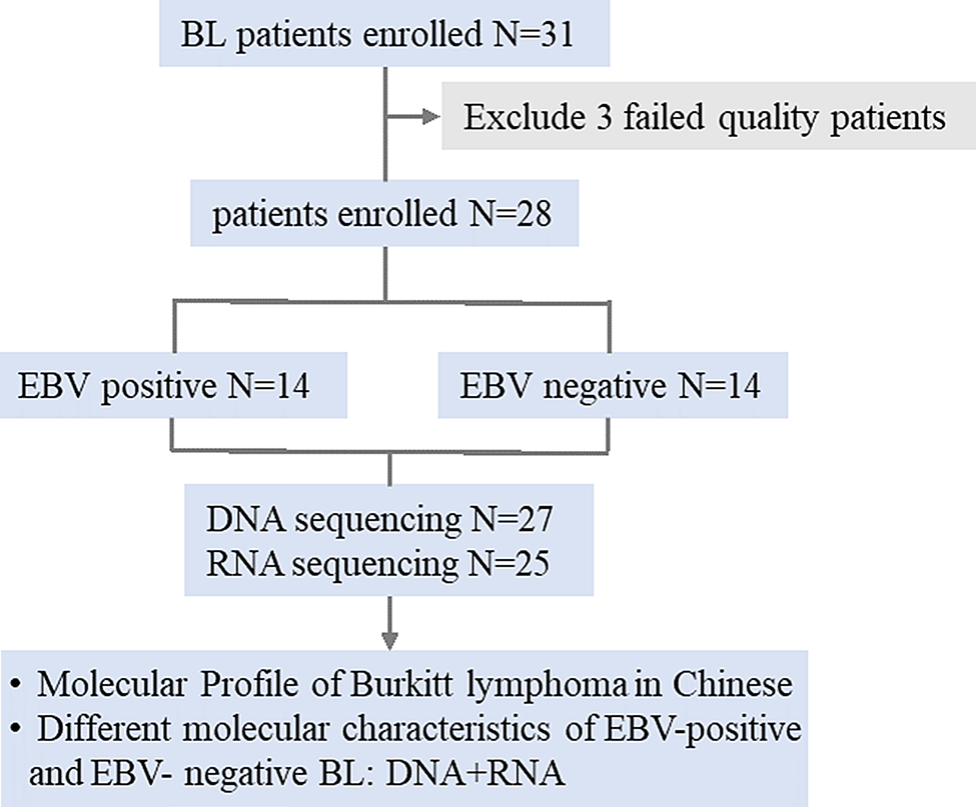


Tissue samples were collected from 31 patients with confirmed BL at The Third Xiangya Hospital between January1, 2015, to December 1, 2023. Baseline clinical and pathological data was obtained from patient medical records. Overall survival (OS) was defined as by the time from the date of diagnosis to the date of the last follow-up or death.

We performed targeted high-throughput sequencing on DNA using a 475-gene panel, and conducted whole transcriptome sequencing (RNA-seq) on the RNA samples. Three samples were excluded as both their DNA and RNA results failed quality control, leaving a total of 28 samples for the study. Twenty-five samples had high-quality RNA-seq data suitable for transcriptome analysis, 27 samples had DNA sequencing data that passed QC for genomic analysis. Twenty-three patients had both DNA and RNA sequencing data passing QC, allowing for integrated genomic and transcriptomic analyses. The details are displayed in Supplemental Table 1.

### Clinical information and EBV status determination

Clinical data of the patients were collected from their medical records, and their baseline information is summarized in Table 1. This study included 28 patients with BL; all samples were classified according to EBV status (Table 1). Fourteen were EBV-negative and 14 were EBV-positive. Males accounted for a higher proportion (75%) than females (25%), and 60.7% of the patients were adults. In Situ Hybridization (ISH) was used to detect EBV-encoded small RNA (EBER) within tumor cells. A negative EBER-ISH result indicates the absence of detectable EBV within the tumor cells, classifying the case as EBV-negative BL, irrespective of the patient’s systemic serological status [18].

**Table 1:** Demographic and characteristics of the patients

		Overall	EBV-	EBV+	*p*
		*N* = 28	*N* = 14	*N* = 14	
Sex (%)	female	7 (25.00)	2 (14.29)	5 (35.71)	0.3827
	male	21 (75.00)	12 (85.71)	9 (64.29)	
Age (median [IQR])		39.5 [6.8, 58.0]	22.5 [6.5, 57.7]	47.5 [14.3, 57.8]	0.4477
Age (%)	Adult (≥ 18y)	17 (60.71)	7 (50.00)	10 (71.43)	0.439
	Pediatric and Teenager	11 (39.29)	7 (50.00)	4 (28.57)	
primary site (%)	head and neck	14 (50.00)	6 (42.86)	8 (57.14)	0.7055
	Non-head and neck	14 (50.00)	8 (57.14)	6 (42.86)	
Status (%)	living	18 (69.23)	9 (69.23)	9 (69.23)	1
	deceased	8 (30.77)	4 (30.77)	4 (30.77)	

### Bulk-RNA sequencing and analysis

Total RNA was extracted from samples using the RNeasy FFPE kit (QIAGEN). Ribosomal RNA was depleted using RNase H, followed by library preparation using the KAPA Stranded RNA-seq Kit with RiboErase (HMR) (KAPA Biosystems). Library concentration was determined by the KAPA Library Quantification Kit (KAPA Biosystems), and library quality was accessed by the Agilent High Sensitivity DNA kit on a Bioanalyzer 2100 (Agilent Technologies). The library was then sequenced on an Illumina HiSeq NGS platform (Illumina).

Base calling was performed on bcl2fastq v2.16.0.10 (Illumina) to generate sequence reads in FASTQ format (Illumina 1.8 + encoding). Differential expression analysis was conducted by R packages DESeq2 (version 1.16.1) and edgeR (version 3.18.1). Differentially expressed genes were selected by Fold Change > 2 and P value < 0.05.

Corresponding volcano plots and heatmaps were generated by in-house R scripts. GO and KEGG enrichment analysis were performed by ClusterProfiler (version 3.4.4). The CIBERSORT16 and xCell algorithm was employed to quantify the proportions and distributions of tumor-infiltrating immune cells based on the RNA-sequencing data. xCell provides three integrated metrics: ImmuneScore, StromaScore, and MicroenvironmentScore, which summarize the overall abundance of immune cells, stromal components, and the combined non-tumor microenvironment, respectively. These scores are derived from spillover-compensated enrichment scores based on immune and stromal cell-type–specific gene signatures.Gene set enrichment analysis (GSEA) was performed using GSEA software (V.4.1.0).

### DNA sequencing

Tumor genomic DNA was extracted from tumor biopsies with QIAamp DNA FFPE Tissue Kit (QIAGEN) following the manufacturer’s instructions. Libraries were constructed using the KAPA Hyper DNA Library Prep Kit (KAPA Biosystem). At last dual-indexed sequencing libraries were PCR amplified with KAPA HiFi Hot start-ready Mix (KAPA Biosystems) for 4–6 cycles, then cleaned up by purification Beads (Corning, AxyPrep Fragment Select-I kit, 14223162).The enriched libraries were sequenced on HiSeq 4000 NGS platforms (Illumina) to coverage depths of 1000×. Trimmomatic was used for FASTQ file quality control.

Single nucleotide variants (SNVs) and short insertions/deletions (indels) were identified by VarScan2 2.3.9 with minimum variant allele frequency threshold set at 0.01, and p-value threshold for calling variants set at 0.05 to generate Variant Call Format (VCF) files. All SNVs/indels were annotated with ANNOVAR, and each SNV/indel was manually checked on the Integrative Genomics Viewer (IGV). Copy number variations (CNVs) were detected using in-house-developed software.

### Statistical methods

Fisher’s exact test was used for discrete variables, while the Wilcoxon rank-sum test and Student’s t-test were applied to continuous variables. Overall survival (OS) was estimated using the Kaplan-Meier method. Hazard ratios were assessed using the Cox proportional hazards model, and comparisons were performed with the Log Rank test. Statistical significance was set at set at a two-sided p-value < 0.05.

## Results

### Screening key gene mutations in BL

**Fig. 1:**
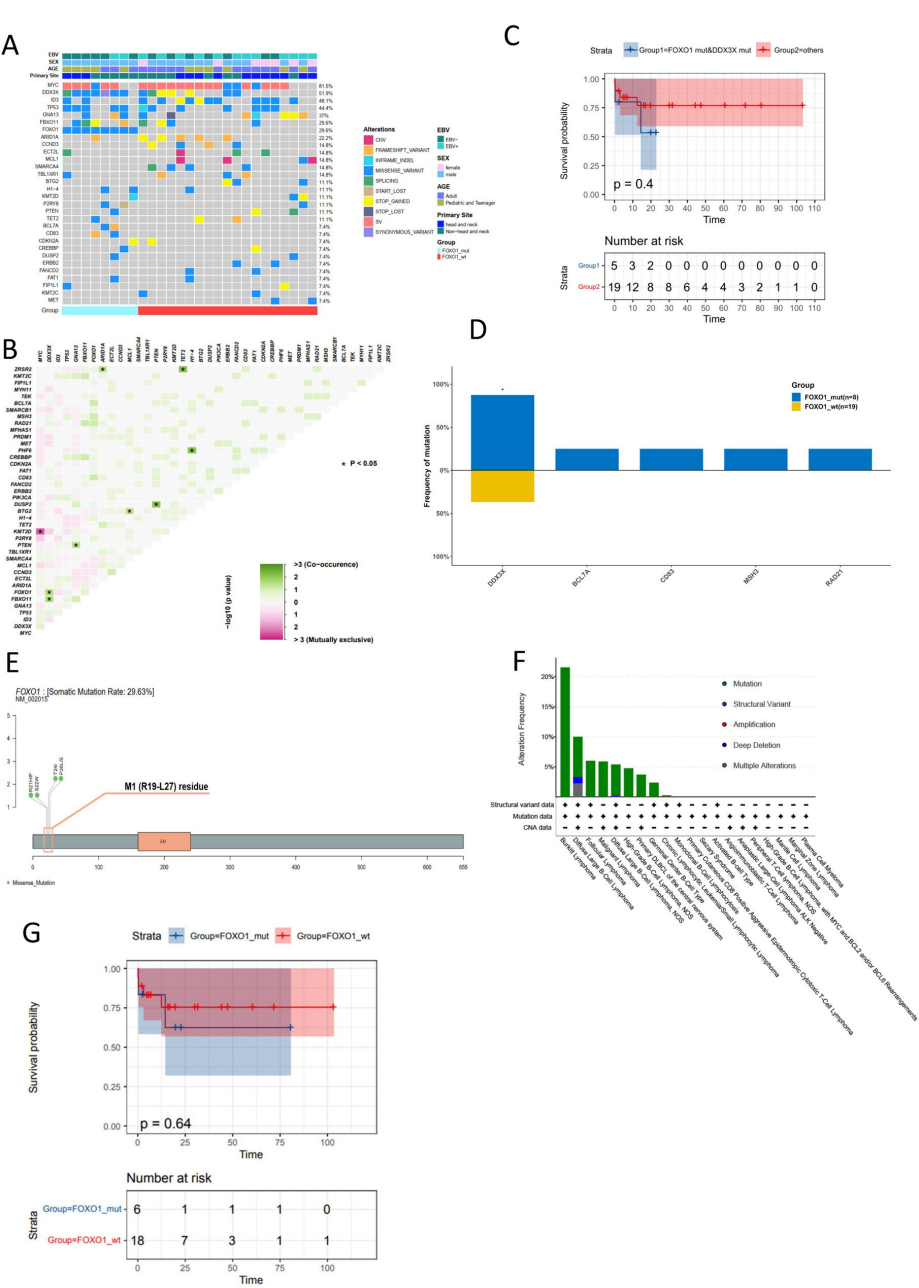
475 Target DNA sequencing in BL to screen mutations. (**A**) Oncoprint shows gene mutations and copy number alteration (CNA) characteristics distributed by case. Each column corresponds to a case, and the right-side histogram uses different colors to represent different types of genetic alterations, with the length of the color bar indicating the number of driver mutations. Each row in the plot represents a gene. Only the top 30 genes by mutation frequency are displayed. (**B**) After removing genes with a mutation frequency below 5%, the mutual exclusivity and co-occurrence of mutated genes in BL patients were evaluated. Green represents co-occurrence, while red represents mutual exclusivity. The depth of the color indicates the degree of interaction between the two genes. (**C**) Survival analysis based on the status of FOXO1 and DDX3X grouping (**D**) The mutation frequency of different genes in FOXO1 wild-type and mutant cases. (**E**) The schematic diagram illustrates the FOXO1 mutation hotspots in the cohort. (**F**) The mutation frequency of FOXO1 across various cancer types in the cBioPortal database. (**G**) Kaplan-Meier curves showing the survival outcomes in patients with FOXO1 wild-type and mutant groups. Asterisks in this figure indicate significant differences according to the adjusted Fisher’s exact test (*P* < 0.05)

To identify the genetic alteration characteristics in BL, we collected tissue samples from 31 confirmed BL patients for DNA extraction. After quality assessment, 4 samples were excluded due to inadequate DNA quality. Consequently, we performed targeted high-throughput sequencing on DNA samples from 27 individuals using a panel of 475 genes. At the genomic level, high-frequency gene mutations in BL include *MYC* (81.5%), *DDX3X* (51.9%), *ID3* (48.1%), and *TP53* (44.4%). *MYC* mutations were detected in 81.5% of cases (22/27), predominantly as missense mutations or rearrangements (Fig. 1A). We excluded genes with mutation frequencies below 5% to assess gene mutual exclusivity and co-occurrence in BL patients (Fig. 1B**).** We found mutual exclusivity between *KMT2D* and *MYC* mutations, and concurrent mutations between *FOXO1* and *DDX3X*, *FBXO11* and *DDX3X*, *PTEN* and *GNA13*, among others (Fig. 1B). Survival analysis based on *FOXO1* and *DDX3X* status grouping revealed a trend towards poorer survival in patients with co-mutations of *FOXO1* and *DDX3X*, although statistical significance was not reached (Fig. 1C). To comprehensively evaluate the clinical and genetic implications of *FOXO1* mutations in our cohort, we stratified patients into *FOXO1*-mutant and wild-type groups. Although *BCL7A*, *CD83*, *MSH3*, *RAD21*, *FBXO11*, *ARID1A*, and *TP53* exhibited relatively higher mutation frequencies in the FOXO1-mutant group, only DDX3X demonstrated a statistically significant difference in the inter-group comparative analysis. The frequency of *DDX3X* mutations was higher in the *FOXO1* mutation group, corroborating the earlier analysis that identified co-occurring mutations between *DDX3X* and *FOXO1* genes (Fig. 1D).

Moreover, our DNA sequencing data revealed *FOXO1* mutations in 8 samples, involving 6 amino acid changes at 5 positions (21–26) (Table 2). These 6 amino acid changes are all located within the M1 residue (Fig. 1E). These mutations may be associated with the uncoupling of FOXO1 from BCR/PI3K signaling pathways, which are known to be involved in promoting proliferation and survival [19]. In the cBioPortal database, out of 334 BL cases, *FOXO1* mutations including missense and nonsense mutations (truncating mutations) were found in 78 patients. Consistent with previous reports in sporadic BL (~ 29%, 23/78) [19], we observed a comparable *FOXO1* mutation frequency of 29.6% in our patient cohort (Fig. 1F). Prompted by studies suggesting a role for *FOXO1* mutations in BL relapse, we performed a comparative analysis of overall survival based on *FOXO1* mutation status. Although no statistically significant difference in survival was observed between the groups, the *FOXO1*-mutated group showed a trend towards poorer survival (Fig. 1G). These findings suggest a potential role of *FOXO1* mutations in BL progression, warranting further validation in larger cohorts.

**Table 2:** FOXO1 gene mutation in the BL cohort

Sample	Type	Gene	AAChange	Ref	Alt	ExonicFunc	AF	EBV Group	Age Group
1	Mutant	FOXO1	c.62G > A (p.R21H)	C	T	missense_variant	26.92%	EBV+	Adult
2	Mutant	FOXO1	c.62G > C (p.R21P)	C	G	missense_variant	74.03%	EBV+	Pediatric
3	Mutant	FOXO1	c.65 C > G (p.S22W)	G	C	missense_variant	30.10%	EBV+	Pediatric
4	Mutant	FOXO1	c.71 C > T (p.T24I)	G	A	missense_variant	39.95%	EBV+	Adult
5	Mutant	FOXO1	c.71 C > T (p.T24I)	G	A	missense_variant	16.88%	EBV-	Pediatric
6	Mutant	FOXO1	c.76 C > T (p.P26S)	G	A	missense_variant	38.78%	EBV+	Pediatric
7	Mutant	FOXO1	c.77 C > T (p.P26L)	G	A	missense_variant	41.10%	EBV+	Pediatric
8	Mutant	FOXO1	c.77 C > T (p.P26L)	G	A	missense_variant	47.94%	EBV+	Pediatric

*EBV+: represent EBV-positive; EBV-: represent EBV-negative;

### The genetic alterations in EBV-positive and EBV-negative BL patients

**Fig. 2:**
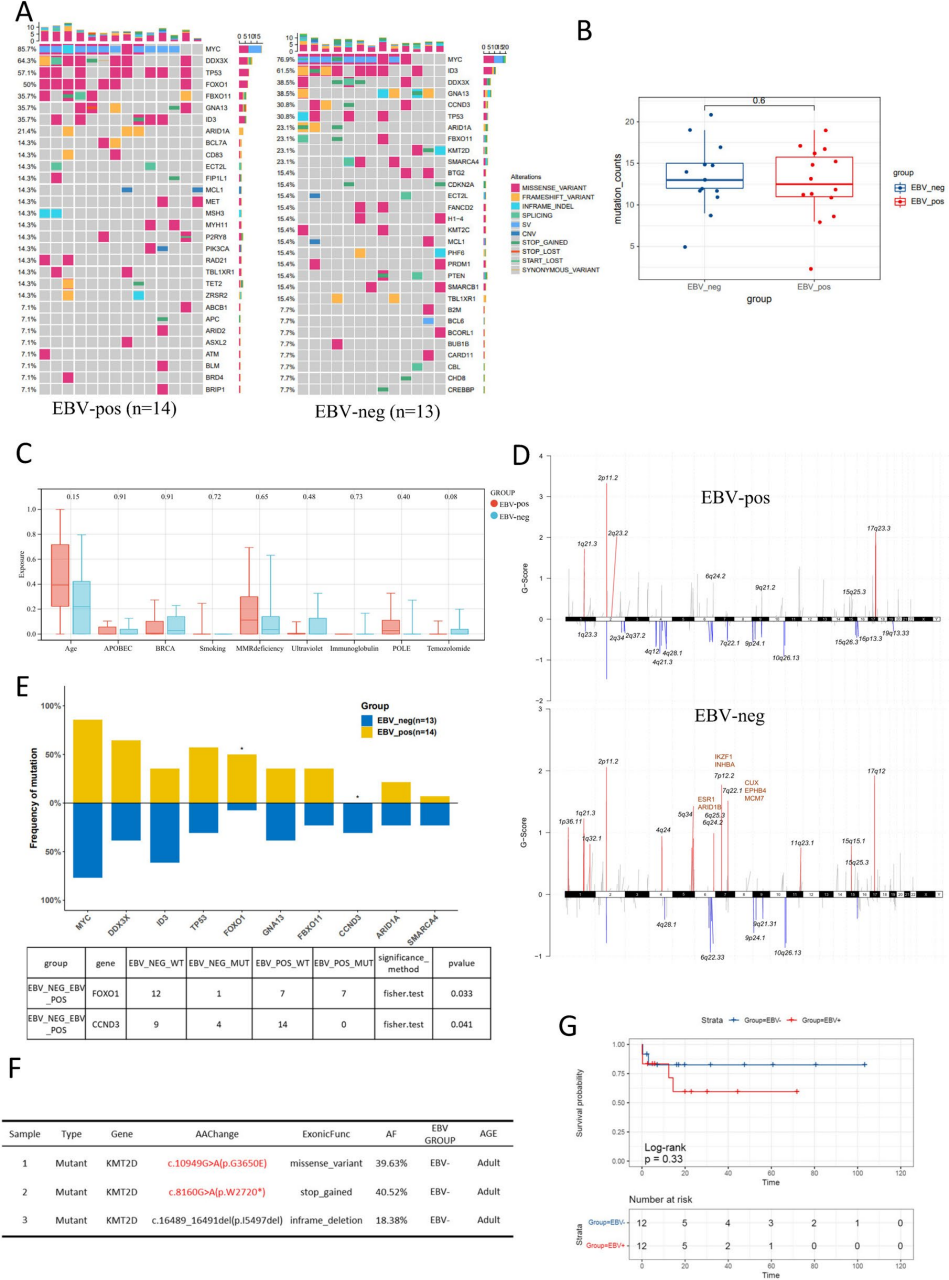
EBV infection and genetic alterations in BL. (**A**) The oncoprint displays the genetic mutation and copy number variation (CN) features for EBV-positive and EBV-negative BL cases. Each column in the figure represents a case, and each row corresponds to a gene and its mutation frequency. Different colors represent different types of genetic alterations, and only the top 30 genes by mutation frequency are shown. (**B**-**C**) The difference in genetic mutation numbers, EBV-positive group showing (**B**) and genetic mutation signatures (**C**) in EBV-positive and EBV-negative BL patients. (**D**) GISTIC analysis of genomic copy number variations in the EBV-positive and EBV-negative groups. (**E**) Differential gene expression analysis based on EBV status grouping. (**F**) Mutation information of KMT2D in EBV-negative samples. (**G**) Survival analysis based on EBV grouping. Asterisks in this figure indicate significant differences according to the adjusted Fisher’s exact test (*P* < 0.05)

To investigate the molecular divergence between EBV-positive and EBV-negative BL, we stratified 27 BL cases based on EBV status and analyzed their genomic profiles (Fig. 2A). We found no significant difference in the total mutation burden between EBV-positive and negative BL patients (Fig. 2B). However, mutational signature analysis demonstrated distinct patterns: EBV-positive tumors exhibited a higher proportion of age-related signatures, while EBV-negative tumors showed enrichment of UV-associated signatures, though these differences did not reach statistical significance (*p* = 0.17 for Age; *p* = 0.11 for Ultraviolet) (Fig. 2C).

GISTIC analysis identified significant genomic disparities between the groups. EBV-negative tumors displayed a higher frequency of chromosomal amplifications, particularly at 7p12.2, 6q25.3 and 7q22.1 (Fig. 2D). We discovered that several identified amplified segments cover well-known oncogenic driver genes, such as *ESR1* (6q25.3), *ARID1B* (6q25.3), *IKZF1* (7p12.2), *CUX* (7p12.1). In addition, the results revealed a significantly higher mutation frequency of *FOXO1* in the EBV-positive group, while *CCND3* showed a higher mutation frequency in the EBV-negative group (Fig. 2E). It is noteworthy that two novel *KMT2D* variants were identified in adult patients in the EBV-negative group: *KMT2D* c.10949G > A (p.G3650E) and *KMT2D* c.8160G > A (p.W2720*) (Fig. 2F). Survival analysis of 24 patients with complete follow-up data demonstrated a non-significant trend toward improved overall survival (OS) in the EBV-negative group (HR = 2.29, 95% CI 0.42–12.51, log-rank *p* = 0.33; Fig. 2G). Further exploration is needed to determine whether these two novel *KMT2D* mutations have specific functional implications in EBV-negative BL progression.

### Transcriptional divergence and prognostic implications in EBV-positive and negative BL

**Fig. 3:**
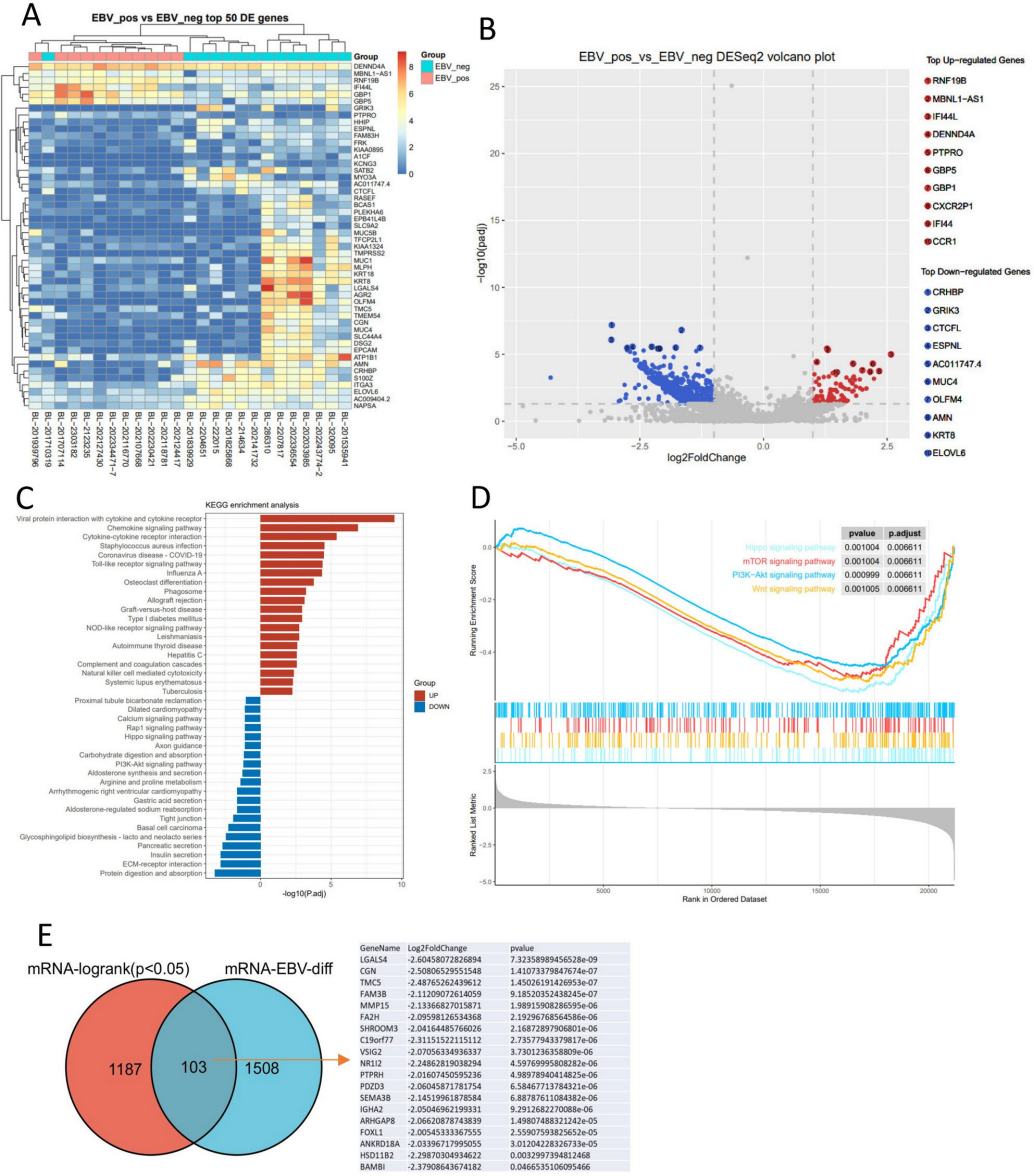
RNA sequencing for EBV-positive and negative BL samples. (**A**-**B**) Heatmap (**A**) and volcano plot (**B**) showing differentially expressed genes in EBV-positive and negative BL samples, with |FC| >2 and p-value < 0.05. (**C**-**D**) KEGG analysis (**C**) and GSEA (Gene Set Enrichment Analysis) (**D**) of pathway enrichment for differentially expressed genes. (**E**) Venn diagram of significantly differentially expressed genes between the EBV-positive and negative groups, highlighting mRNAs that are associated with survival

To delineate the transcriptional landscape underlying EBV-associated BL, we performed RNA sequencing on 25 BL cases (11 EBV-positive, 14 EBV-negative) and identified 1,612 DEGs between groups (|log2FC| >1, FDR < 0.05; Fig. 3A-B). EBV-positive BL demonstrates a distinct transcriptional profile compared to EBV-negative cases, marked by pronounced upregulation of *RNF19B*, *MBNL1-AS1*, *IFI44L*, *DENND4A*, *PTPRO*, and significant downregulation of *CRHBP*, *GRIK3*, *CTCFL*, *ESPNL*, and *AC011747* (Fig. 3A-B). Significantly downregulated genes in EBV-positive tumors were enriched in PI3K-AKT and Hippo signaling pathways (KEGG, *p* < 0.05; Fig. 3C). In GSEA enrichment analysis, important tumor signaling pathways including PI3K-AKT, Hippo, WNT, and mTOR were significantly downregulated in the EBV-positive group (FDR < 0.05; Fig. 3D).

Univariate survival analysis (Log-rank test) of dichotomized mRNA expression data revealed 1,290 significant transcripts. Cross-referencing these with the DEGs defined by EBV status revealed a set of 103 genes significantly linked to both survival and EBV infection, all of which were down-regulated in EBV-positive tissues (Fig. 3E).

### EBV-positive BL exhibits enhanced M1 macrophage infiltration linked to immune-active microenvironment

**Fig. 4:**
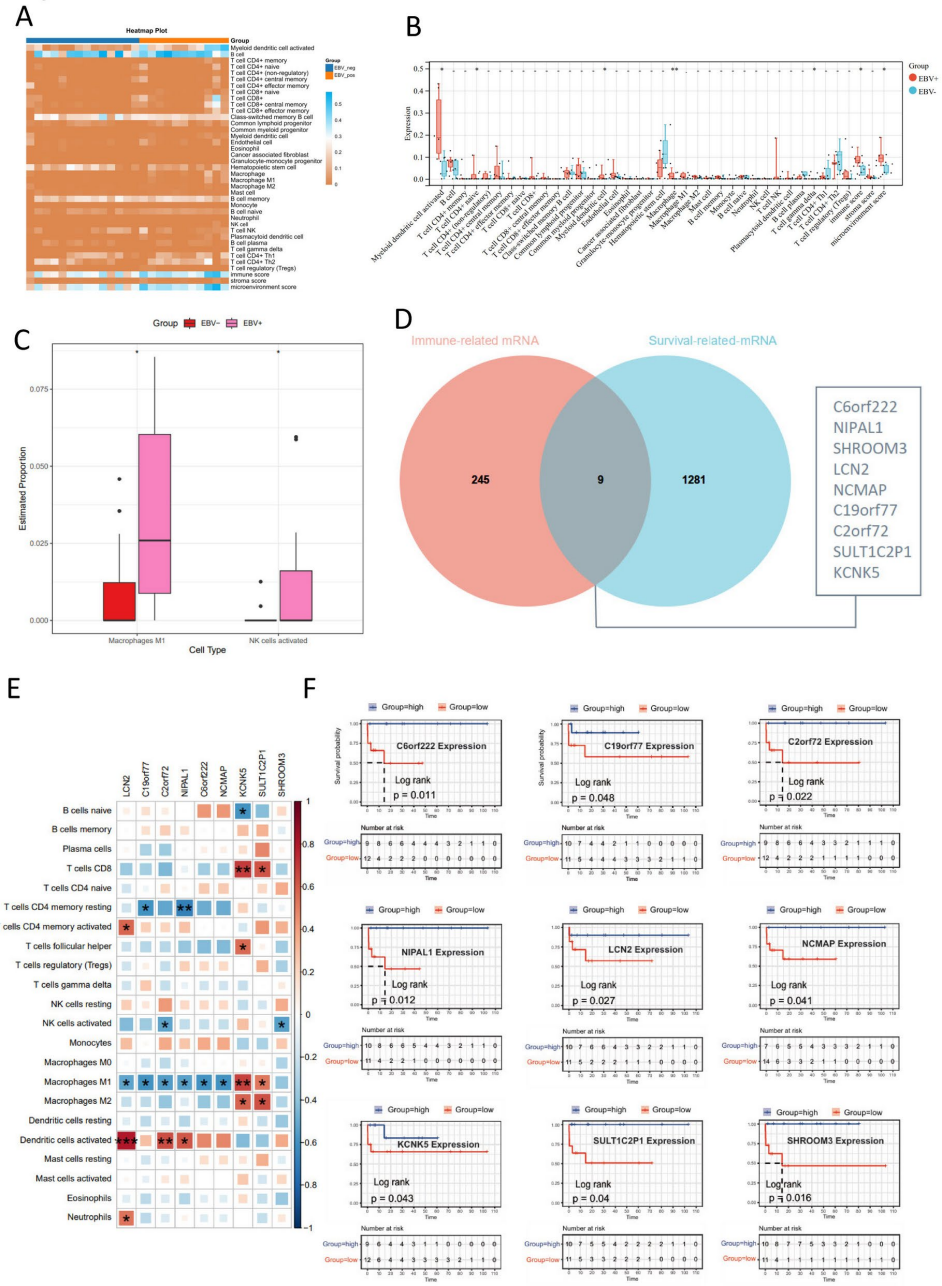
Differential immune infiltration in EBV-positive and negative BL samples. (**A**) Heatmap showing immune cell infiltration in EBV-positive and EBV-negative samples. (**B**) Differential immune cell analysis between groups based on EBV status. (**C**) Analysis of macrophage M1 and activated NK cell infiltration abundance in different EBV groups using the CIBERSORT method. (**D**) Venn diagram of genes significantly correlated with macrophage M1 and NK immune cells, and mRNAs associated with prognosis. (**E**) The correlation between immune cell infiltration abundance and gene expression. (**F**) Survival curves of high and low expression groups for 9 immune-related differentially expressed genes. Asterisks in this figure indicate significant differences according to the *p* < 0. 05

To date, few studies have focused on the role of the tumor microenvironment in BL. In this study, we systematically investigated the immune cell infiltration patterns in EBV-positive and negative BL based on their differential gene expression profiles. Using the xCELL algorithm, we identified 36 immune cell subtypes significantly associated with the tumor immune microenvironment. Among these, activated myeloid dendritic cell, granulocyte-monocyte progenitor, macrophage, and both M1 and M2 macrophage subtypes showed significantly higher infiltration abundances in the EBV-positive group compared to the EBV-negative group. Additionally, immune score and stromal score were significantly higher in the EBV-positive group (Fig. 4A-B). Further, we conducted immune infiltration analysis using the CIBERSORT method and found significantly higher infiltration abundances of M1 macrophages and activated NK cells in the EBV-positive group compared to the EBV-negative group (Fig. 4C). Both xCELL and CIBERSORT immune infiltration analyses consistently showed significant differences in M1 macrophages between the groups. Spearman correlation analysis identified 254 genes associated with M1 macrophages, intersecting with survival-associated mRNA (log-rank *p* < 0.05 for 1,290 genes). Nine hub genes showed dual associations with M1 infiltration and patient prognosis (Fig. 4D). The nine hub genes showed significant downregulation in the EBV-positive group. Correlation analysis between immune infiltration results and these 9 genes revealed that the expression of *C6orf222*, *NIPAL1*, *SHROOM3*, *LCN2*, *NCMAP*, and *C19orf77* genes was negatively correlated with M1 macrophage infiltration, while *SULT1C2P1* and *KCNK5* expression positively correlated with M1 macrophages (Fig. 4E**).** Macrophages play a crucial role in tumorigenesis, regulation of angiogenesis, immune suppression, and metastasis. High infiltration of macrophages in tumors is also associated with poor cancer prognosis. Therefore, we next analyzed the impact of the expression of these 9 genes on the prognosis of BL, respectively. The results showed that the high-expression group of these 9 genes exhibited better survival compared to the low-expression group (Fig. 4F).

### Differential expression of LncRNAs between EBV-positive and negative groups

**Fig. 5:**
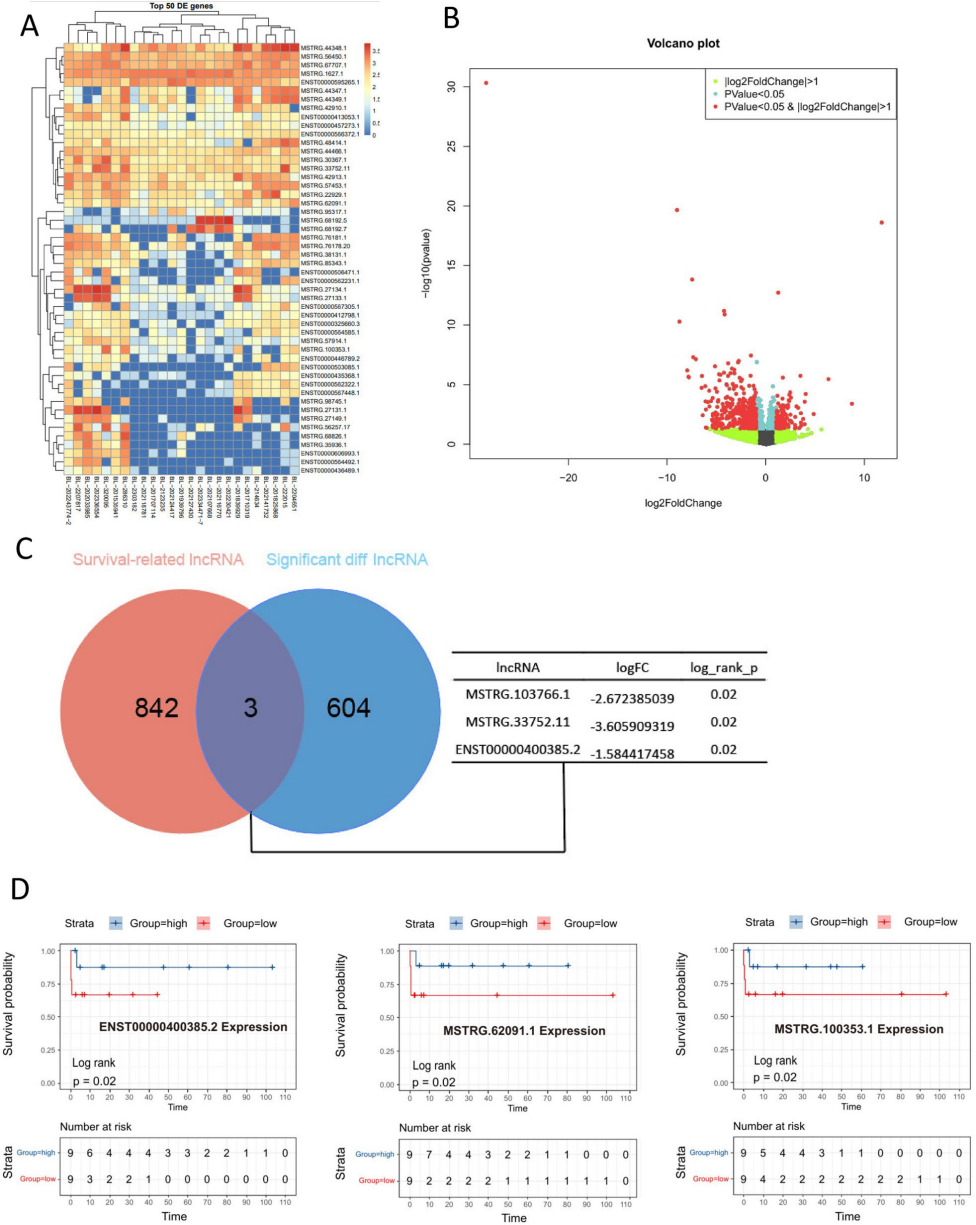
lncRNA analysis in EBV-positive and negative groups. (**A**-**B**) Heatmap (**A**) and volcano plot (**B**) showing the differentially expressed lncRNAs between the EBV-positive and the EBV-negative group. (**C**) The intersection of prognostic-related lncRNAs and differentially expressed lncRNAs based on EBV grouping. (**D**) Kaplan-Meier survival curves revealed a significant correlation between elevated expression of these three lncRNAs and adverse prognostic outcomes in BL

Small RNAs also play a crucial role in tumor progression [20], so we analyzed the expression of lncRNAs in EBV-positive and negative BL. Differential analysis identified 607 significantly dysregulated lncRNAs (|log2FC| >1, *p* < 0.05), including 125 upregulated and 482 downregulated transcripts in EBV-positive tumors (Fig. 5A-B). Among these, three downregulation lncRNAs (MSTRG.103766.1, MSTRG.33752.11, ENSTO0000400385.2) in EBV-positive cases showed strong prognostic value (Fig. 5C). Kaplan-Meier analysis demonstrated that high expression of each of these three lncRNAs was significantly associated with longer overall survival in patients with BL (Fig. 5D). Thus, we have uncovered a set of prognostically adverse lncRNAs downregulated in EBV-positive BL, suggesting they may represent functionally significant players in EBV-driven lymphomagenesis.

## Discussion

EBV infection is common in BL, yet its mechanistic role in shaping the genomic and immune landscape of BL remains poorly characterized. Our study provides a comprehensive multi-omics analysis comparing EBV-positive and negative BL, revealing distinct molecular signatures that may underlie EBV-driven lymphomagenesis and therapeutic responses.

A pivotal finding of this work is the identification of recurrent FOXO1 mutations in EBV-positive BL. *FOXO1* (Forkhead box O1) ,a member of the FOX transcription factor family, plays a crucial role in various biological processes, including cell proliferation, apoptosis, metabolism, and antioxidant responses [21]. It exhibits multifunctional roles in development and immunity, demonstrating both tumor-suppressive and oncogenic capabilities, depending on the cellular context. Human B cell lines carrying *FOXO1* mutations show overactivation of PI3K and SAPK/JNK signaling pathways [22]. Additionally, these mutations can alter FOXO1’s transcriptional affinity, promoting transcriptional programs associated with germinal center positive selection and enhancing cell survival [22].

Previous studies have reported that mutations affecting residues M1 (R19-L27) in diffuse large B-cell lymphoma (DLBCL) lead to the loss of the AKT recognition motif or abnormal nuclear localization of FOXO1, correlating with poorer prognoses [19]. Activating *FOXO1* mutations have also been identified in mouse models of MYC/PI3K-driven BL and human BL cell lines [22]. These models demonstrate that the T24 mutation in *FOXO1* disrupts coupling with BCR/PI3K, driving nuclear localization of FOXO1 protein and promoting proliferation and survival. Our DNA sequencing data reveals *FOXO1* mutations occurred in 8 samples, involving 6 *FOXO1* variants clustered within the M1 domain (residues 21–26), a region critical for AKT-mediated cytoplasmic sequestration. Among these, c.65 C >G (p.S22W) has been reported in DLBCL [23], and c.71 C >T (p.T24I) has been reported in BL [19]. The other 4 variants have been detected in pediatric BL [19], but there are no functional reports available. Whether these four mutations have functional significance in BL requires further experimental validation. The six amino acid changes discovered in this study are all located within the M1 residue, their spatial overlap with known activating mutations strongly suggests pathogenic relevance. These mutations may be associated with the uncoupling of *FOXO1* from BCR/PI3K signaling pathways, which are known to be involved in proliferation and survival promotion in BL [24]. Based on our finding that 87.5% (7 out of 8) of patients with *FOXO1* mutations are EBV-positive, we consider that *FOXO1* may be a key mutation driven by EBV infection and could be a crucial factor in the progression of EBV-positive BL.

In addition to the distinct mutational profile of *FOXO1*, our genomic analysis also identified recurrent mutations in the epigenetic modulator *KMT2D*, particularly within the EBV-negative subgroup. KMT2D is a histone methyltransferase responsible for catalyzing the methylation of histone H3 lysine 4 (H3K4). Its mutation or functional loss may lead to reduced H3K4 methylation levels, dysregulated gene expression, and promote abnormal proliferation of lymphoma cells and tumor progression [25]. In acute leukemia (AML), KMT2D has been demonstrated to regulate ribosomal biogenesis by modulating the mTOR signaling pathway, thereby influencing tumorigenesis and progression [25]. Our study data similarly revealed multiple-site mutations of *KMT2D* in EBV-negative BL, along with a mutually exclusive mutation pattern between *KMT2D* and *MYC*. Further RNA sequencing data showed that up-regulated genes in the EBV-negative group were enriched in mTOR and other signaling pathways. Based on literature reports and our experimental results, these findings suggest that *KMT2D* mutations may be closely associated with tumor progression in EBV-negative BL.

*KMT2D* is one of the most frequently mutated genes in diffuse large B-cell lymphoma (DLBCL) [26], with mutations predominantly occurring in the SET domain and PHD domain, which are the primary functional regions of the KMT2D enzyme [27, 28]. Since *KMT2D* mutations are often nonsense or frameshift mutations, these mutations will lead to KMT2D functional loss and reduced H3K4 methylation. As a prognostic biomarker and therapeutic target, the mutation status of *KMT2D* can serve as an indicator for prognosis assessment in lymphoma patients, with its mutations being associated with advanced disease, relapse risk, and poor prognosis. Furthermore, studies have shown that *KMT2D* mutations can alter the recruitment and function of regulatory T cells (Treg) in the tumor microenvironment. In diffuse large B-cell lymphoma (DLBCL), *KMT2D* mutations promote the migration of Treg cells into the tumor microenvironment and suppress immune responses through the FBXW7-NOTCH-MYC/TGF-β1 signaling axis, thereby promoting tumor growth [29]. In our study, we identified three *KMT2D* gene mutations in EBV-negative BL, including a frameshift mutation at position 5497 of KMT2D located in the SET domain, and two other mutations at positions 3650 and 2720, which are outside the hotspot mutation regions. The functional significance of these two novel KMT2D mutations in EBV-negative BL progression remains to be determined and warrants further experimental validation in future studies.

EBV facilitates tumor immune evasion by modulating PD-L1, NK cell checkpoints, HLA expression, and cytokine networks. Simultaneously, its latent proteins (e.g., LMP1, EBNA1) and non-coding RNAs upregulate immunosuppressive molecules through STAT3, NF-κB, and other signaling pathways while suppressing antigen presentation [30]. Whether EBV-positive BL exhibits a higher propensity for immune escape warrants further investigation. BL is characterized by a tumor microenvironment (TME) where macrophages are a predominant component, contributing to a distinctive histological appearance known as the “starry sky” pattern [31]. Macrophages play a crucial role in tumor initiation, angiogenesis, immune suppression, and metastasis [32–34]. High infiltration of macrophages in tumors is also associated with poor cancer prognosis [35]. Besides, the mutations of *FOXO1* or *KMT2D* are demonstrated closely related to tumor microenvironment. However, current research on the role of the immune microenvironment in BL progression remains remarkably scarce. In our study, we found that macrophages, including M1 and M2 types, myeloid dendritic cells, and granulocyte-monocyte progenitor cells were significantly more abundant in the EBV-positive group compared to the EBV-negative group. Several studies have found differences in immune response characteristics between BL with granulomatous reactions and typical “starry sky” pattern BL (whether EBV-positive or EBV–negative). BL with diffuse granulomatous reactions exhibits a pro-inflammatory TME with M1–Th1 polarization, associated with controlled tumor growth and spontaneous remission. In contrast, the “starry sky” pattern of BL shows M2 polarization and upregulated pro-tumor responses [31, 36]. In our study, none of the cases exhibited granulomatous reactions. Future experiments should explore whether differences in infiltrating immune cells between EBV-positive and EBV–negative groups affect tumor growth and progression.

Although our RNA-seq data identified upregulated expression of *RNF19B*, *MBNL1-AS1*, *IFI44L*, *DENND4A*, and *PTPRO* in EBV-positive BL tissues, the biological functions of these genes in BL remain to be elucidated. Among these five genes, only *IFI44L* has been previously reported in gastric cancer, where its high expression is associated with EBV infection [37]. The relationship between EBV status and the expression of the other four genes, however, remains entirely unexplored. Furthermore, our analysis identified a cohort of 103 mRNAs that reside at the critical intersection of EBV-associated dysregulation and patient survival. While the functional characterization of each individual gene falls beyond the scope of this study, this set constitutes a high-priority candidate library for future investigation. The convergence of differential expression and prognostic significance strongly implies that these genes are embedded in the core mechanistic pathways through which EBV influences BL pathogenesis and clinical outcomes. Future studies aimed at validating these candidates, through functional genomics in relevant models or correlation with protein expression, are warranted to dissect their precise roles as drivers, passengers, or mediators of the distinct immune microenvironment in EBV-positive BL. Beyond protein-coding genes, our study also revealed a landscape of dysregulated lncRNAs in EBV-positive BL. Research on lncRNAs in BL remains narrowly focused on a few molecules like PVT1 [38]. To expand this understanding, our study employed an integrated multi-omics framework to characterize the global lncRNA regulatory network, pinpointing specific lncRNAs (MSTRG.103766.1, MSTRG.33752.11, ENSTO0000400385.2) as potential key regulators in EBV-associated pathogenesis and prognosis. These three lncRNA are downregulated in EBV-positive cases, while the functional contributions of these lncRNAs to lymphomagenesis are an uncharted territory, demanding further experimental exploration to define their precise roles in tumorigenesis, progression, and drug resistance.

Our study primarily utilized DNA-seq and RNA-seq methods to explore molecular genetic differences between BL with and without EBV infection. We identified a set of genes and signaling pathways associated with EBV infection, providing a molecular foundation for further understanding the role of EBV in the development and progression of BL. However, our study still exists some limitations. Despite being comparable to other genomic studies of BL, a larger cohort would enhance the statistical robustness of our findings, specifically in characterizing inter-patient heterogeneity across clinical subtypes and in definitively analyzing survival differences by EBV status. Secondly, the association between M1 macrophage infiltration and improved survival, identified through computational deconvolution, awaits confirmation through orthogonal methods such as immunohistochemistry on tissue sections. Our integrated multi-omics profiling defines the molecular basis of EBV-driven lymphomagenesis, identifying key alterations in *FOXO1* and *KMT2D* and a unique immune microenvironment, thereby providing actionable targets and testable hypotheses for future mechanistic and therapeutic exploration.

## Supplementary Information

Below is the link to the electronic supplementary material.

Supplementary Material 1

## Data Availability

The raw sequencing data generated during this study are available from the corresponding author upon reasonable request. Researchers may contact chu156@csu.edu.cn for data access inquiries.
